# Imposex in the dogwhelk (*Nucella lapillus*): 22-year monitoring around England and Wales

**DOI:** 10.1007/s10661-015-4961-0

**Published:** 2015-11-10

**Authors:** E. E. Manuel Nicolaus, Jon Barry

**Affiliations:** Centre for Environment, Fisheries and Aquaculture Science, Lowestoft, Suffolk NR33 0LB UK

**Keywords:** TBT pollution, Imposex, *Nucella lapillus*, Marine monitoring, England and Wales

## Abstract

Six imposex surveys in the dogwhelk (*Nucella lapillus*) have been conducted over the past two decades to assess legislation effectiveness controlling the use of tri-butyl tin (TBT) by the maritime shipping industry. This study firstly analysed the results of the 2014 survey and secondly carried out a trend assessment of the same 88 sampled sites between 1997 and 2014 of which 65 showed statistically significant reductions. To highlight the magnitude of change, the Vas Deferens Sequence stages (VDS) of the same 56 sites sampled in 1997 and 2010 showed that the Vas Deferens Sequence Index (VDSI) reduced statistically significantly from 2.89 and 0.42, respectively. These data confirm that the legislation enacted, latterly through the International Maritime Organization (IMO) during 2003 to 2008, has been effective in progressively reducing the impact of TBT in the marine environment. Nevertheless, the 2014 results show that two of 18 sampled sites (Gurnard Bay and St. Mawes) are still above the Ecotoxicological Assessment Criteria (EAC) derived for TBT (VDSI ≤ 2).

**Electronic supplementary material:**

The online version of this article (doi:10.1007/s10661-015-4961-0) contains supplementary material, which is available to authorized users.

## Introduction

Imposex is the imposition of male sexual characteristics on female gastropods and has been found to be one of the most sensitive indicators of environmental tri-butyl tin (TBT) exposure. The incidence of imposex was found to be caused by TBT when used as a biocide in marine antifouling paints for both yachts and large ships in the 1960s (Waldock et al. 1988). In *Nucella lapillus*, the effect is dose related and severe imposex can lead to sterility in females and detrimental reproductive effects on both individuals and populations. The process and characterisation of imposex have been well documented (see, for example, Gibbs et al. 1987; Oehlmann et al. 1991; Santos et al. 2002). Dogwhelk monitoring is also an established biological effect monitoring tool used by many Oslo and Paris Convention (OSPAR 2013a) contracting countries to assess good environmental status by 2020 for the Marine Strategy Framework Directive.

Over the last two decades, legislation has been introduced, firstly to control the use of TBT-based antifouling paints on yachts and, more recently, the prohibition of the use of these biocides on large ships. Imposex (in *N. lapillus*) surveys like those undertaken in 1992 and 1997 for England and Wales were conducted to establish data to support and justify United Kingdom (UK) and European Union (EU) legislation restricting the use of organotin compounds. In 2003, the International Maritime Organization (IMO) introduced legislation banning the use of organotin on all ships. As seagoing vessels may be repainted with antifouling paint only every 5 years, this ban was implemented during 2003–2008. Surveys conducted in 2004, 2007 and 2010 span the period prior to and during the implementation of the IMO worldwide ban (2003–2008) on the use and application of TBT antifouling paints on all commercial shipping, and therefore serve as a time series allowing us to measure the effectiveness of the legislation. The 2014 survey aimed to study the effectiveness of these measures, 6 years after TBT-containing antifouling paints were fully removed from use.

The surveys of 1992, 1997, 2004 and 2007 established a network of 102 sampling sites (Table 1) across England and Wales. The 2007 survey used 86 sites, reduced in number either because dogwhelks had disappeared from some sites or because some sites were too close to each other, i.e. duplicate sampling. The strategy of the 2010 survey was to revisit all of the 2007 sampling sites, while the primary aim of the 2014 survey was to apply a more risk-based approach by only visiting sites that either indicated effects of TBT at or above (OSPAR) assessment class C in the 2010 or 2007 survey, or where the Environment Agency had recorded TBT levels in the water column above the EQS (personal communication with Ali Miles and Graham Phillips from the EA; sites indicated in Table 1).

**Table 1:** All 102 imposex sampling sites and when they were sampled

Stratum	Site name	Latitude	Longitude	1992	1997	2004	2007	2010	2014
Anglia	Dumpton Gap	51.3494	1.442				X		
Anglia	Herne Bay	51.3801	1.2				X		
Anglia	Palm Bay	51.3934	1.4222				X	X	
Anglia	Walpole Bay	51.3938	1.405				X		X
Cardigan Bay	Abermawr	51.9713	−5.0831		X	X	X	X	
Cardigan Bay	Aberystwyth	52.4126	−4.0921		X	X	X	X	
Cardigan Bay	Cable Bay	53.2072	−4.5004		X		X	X	
Cardigan Bay	Caernarfon	53.1403	−4.2845		X				
Cardigan Bay	Church Bay (Anglesey)	53.3709	−4.5577		X		X	X	
Cardigan Bay	New Quay	52.2175	−4.3602		X	X	X		
Cardigan Bay	Porth Colmon	52.8755	−4.6824		X	X	X	X	
Cardigan Bay	Puffin Island	53.3136	−4.0412		X				
Cardigan Bay	Sandy Beach	53.3338	−4.5764		X	X	X	X	
Cardigan Bay	Sarn Bach	52.8116	−4.4987		X		X		
Cardigan Bay	South Stack	53.2999	−4.6793		X		X	X	
Cardigan Bay	Trearddur	53.2072	−4.5004		X		X	X	
Cardigan Bay	Trefor	52.9992	−4.4206		X		X	X	
Eastern Channel	Bembridge	50.6889	−1.0713				X	X	
Eastern Channel	Bill of Portland	50.5125	−2.4577	X	X		X	X	
Eastern Channel	Brixham	50.4004	−3.5052	X	X	X	X	X	
Eastern Channel	East Cowes	50.7663	−1.276				X		X^a^
Eastern Channel	Eastbourne	50.7583	0.2832	X	X	X	X	X	
Eastern Channel	Folkestone	51.0849	1.202	X	X	X	X		
Eastern Channel	Gurnard Bay	50.7602	−1.3254				X	X	X^a^
Eastern Channel	Hanover Point	50.6519	−1.4652				X		X
Eastern Channel	Hayling Island	50.787	−0.9376	X					
Eastern Channel	Horse Ledge Shanklin	50.6186	−1.1714				X	X	X
Eastern Channel	Selsey Bill	50.7297	−0.7761	X	X		X	X	
Eastern Channel	St. Catherine’s Point	50.5758	−1.3				X	X	
Eastern Channel	St. Margaret’s Bay	51.1523	1.3892				X	X	
Eastern Channel	Totland Bay	50.6839	−1.544				X		X^a^
Eastern Channel	Ventnor	50.5919	−1.2118				X		X
Eastern Channel	West Bay	50.7113	−2.7714	X	X	X		X	
Humber/Wash	Sewerby	54.0995	−0.1525	X	X	X	X		
Irish Sea	Cemaes Bay	53.4153	−4.4525		X	X	X	X	
Irish Sea	Cemlyn Bay	53.4136	−4.5145		X				
Irish Sea	Great Ormes Head	53.3252	−3.8604		X		X	X	
Irish Sea	Llanbadrig	53.441	−4.45		X	X		X	
Irish Sea	Llanellen	53.4113	−4.2933		X		X	X	
Irish Sea	Maryport	54.7224	−3.5003		X	X	X		X
Irish Sea	Moelfre	53.353	−4.2346		X		X	X	
Irish Sea	Rhos-on-Sea	53.3085	−3.7367		X	X	X	X	
Irish Sea	Roa Island	54.0726	−3.1735		X	X	X	X	X^a^
Irish Sea	St. Bees Head	54.4898	−3.6122		X	X	X	X	X^a^
Irish Sea	Whitehaven	54.5591	−3.5913		X		X	X	
Severn	Angle Bay	51.688	−5.0754		X		X	X	
Severn	Barry Island	51.3858	−3.2783				X	X	
Severn	Combe Martin	51.2091	−4.0414		X	X	X	X	
Severn	Dale Fort	51.7039	−5.1524		X	X	X	X	
Severn	Freshwater West	51.6536	−5.0611		X	X	X	X	
Severn	Hartland Quay	50.996	−4.5336		X	X	X	X	
Severn	Holywell Bay	50.3906	−5.1477		X				
Severn	Lavernock Point	51.4054	−3.1669		X	X	X	X	
Severn	Limpert Bay	51.3834	−3.4144				X	X	
Severn	Manorbier	51.643	−4.8063		X	X	X	X	
Severn	Marloes Sands	51.7226	−5.2167		X	X	X	X	
Severn	Martin’s Haven	51.7365	−5.2445		X	X	X	X	
Severn	Monk Haven	51.7097	−4.1454		X	X	X	X	
Severn	Penarth	51.3938	−3.1605				X		
Severn	Pennar Jetty	51.6888	−4.9777		X				
Severn	Pennar Point	51.6919	−4.9662		X		X	X	
Severn	Perranporth	50.348	−5.1582		X		X	X	
Severn	Polzeath	50.5743	−4.9216		X	X	X	X	
Severn	Porlock Weir	51.2192	−3.6235		X	X	X	X	
Severn	Port Eynon	51.5408	−4.2063		X	X	X	X	
Severn	Porth Beach	50.426	−5.0628		X	X	X	X	
Severn	Porthcorthan	50.5098	−5.0303		X	X	X	X	
Severn	Porthtowan	50.2871	−5.2454		X	X	X	X	
Severn	Portquin	50.5897	−4.8694		X		X	X	
Severn	Renny’s Slip	51.7324	−5.2447		X	X		X	
Severn	Sennen Cove	50.0785	−5.7034		X	X	X	X	
Severn	Stackpole Quay	51.6241	−4.8999		X	X	X	X	
Severn	Tenby	51.6792	−4.6989		X	X	X	X	X
Severn	Trenance	50.4656	−5.0344		X				
Severn	Trevone	50.5456	−4.981		X				
Severn	Treknow	50.6452	−4.7624		X		X	X	
Severn	Watwick Bay	51.6921	−5.1601		X	X	X	X	
Severn	West Angle Bay	51.6859	−5.1102		X	X	X	X	
Severn	West Pentire	50.4056	−5.131		X				
Severn	Westdale Bay	51.7073	−5.1881		X	X	X	X	
Severn	Whitesands Bay	51.898	−5.2986		X	X	X		
Tyne Tees	Amble	55.3377	−1.5714			X		X	
Tyne Tees	Blyth	55.1306	−1.5001			X	X	X	
Tyne Tees	Blyth Ferry	55.1242	−1.4892	X	X				
Tyne Tees	Boulmer	55.4241	−1.5774	X	X	X	X	X	
Tyne Tees	Hartlepool	54.7	−1.1794			X	X	X	X
Tyne Tees	Parson’s Rock	54.9316	−1.3659				X	X	X
Tyne Tees	Robin Hood’s Bay	54.4232	−0.5277				X	X	
Tyne Tees	Saltburn	54.5869	−0.951				X	X	
Tyne Tees	Scarborough	54.2881	−0.3957				X	X	
Tyne Tees	St. Mary’s Lighthouse	55.0717	−1.4525			X		X	
Tyne Tees	Tees	54.648	−1.1534				X	X	X
Tyne Tees	Tynemouth (Outside)	55.019	−1.4176				X	X	
Tyne Tees	Whitby	54.4907	−0.6093	X	X	X	X	X	X
Tyne Tees	Whitley Bay	55.039	−1.4312				X	X	
Western Channel	Bovisand	50.3349	−4.1208	X	X	X	X	X	X^a^
Western Channel	Gwennap Head	50.0367	−5.6716	X	X	X	X	X	
Western Channel	St. Agnes	50.3222	−5.2043		X				
Western Channel	St. Mawes	50.1554	−5.0251				X	X	X
Western Channel	Start Point	50.2197	−3.6501	X	X	X	X	X	
Western Channel	Swanpool	50.1396	−5.0763				X	X	X
Western Channel	Towan Head	50.1563	−4.984		X		X	X	

^a^Additional 6 sites sampled in 2014 for the Environment Agency

This paper firstly looks at a spatial assessment of the 18 sites sampled in 2014 and secondly examines the results of the 1997 to 2014 surveys with a direct comparison between the same stations sampled more than once between 1997 and 2014.

## Materials and methods

### Sampling sites

**Fig. 1:**
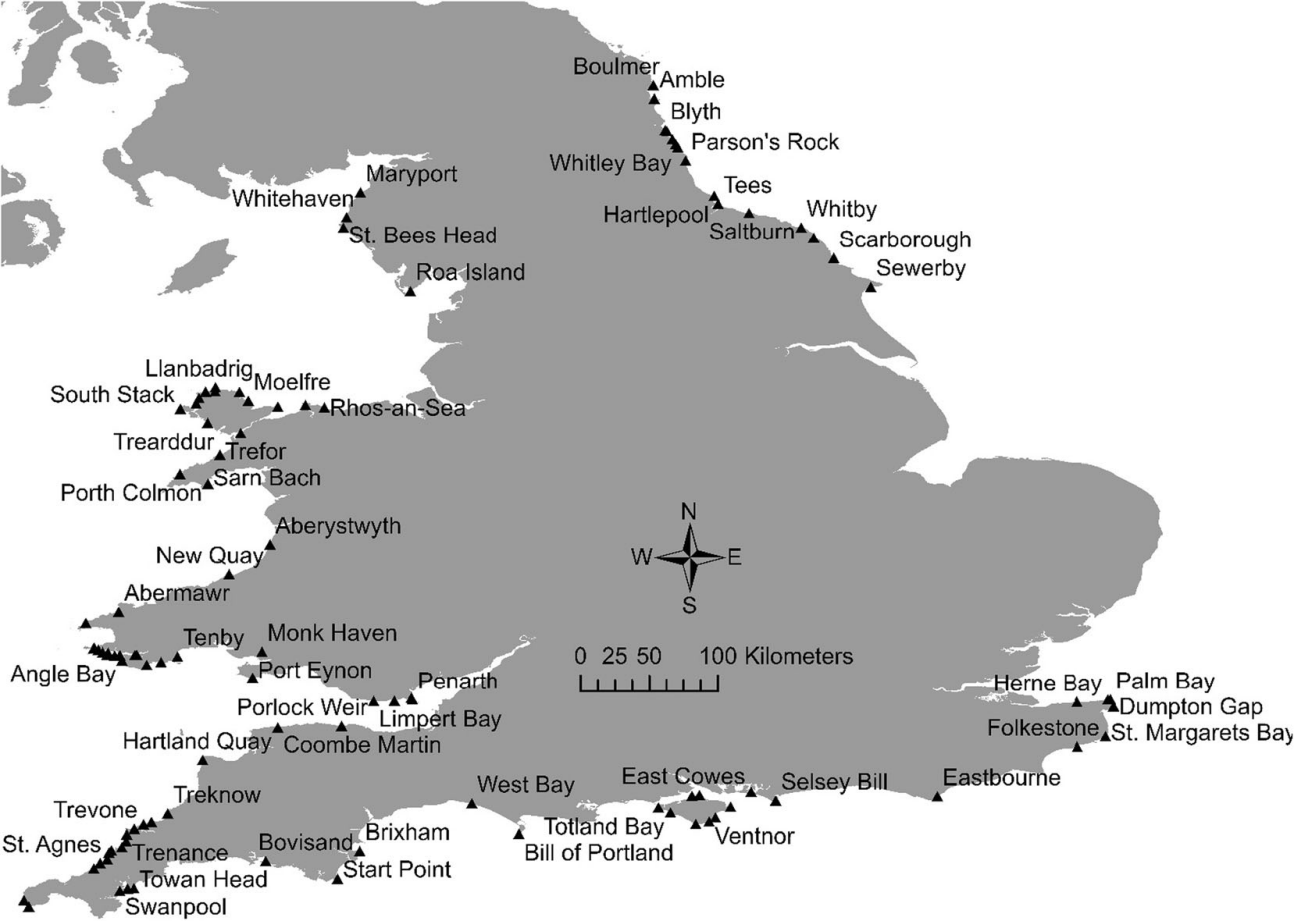
All 102 imposex sampling sites in England and Wales

The aim of the limited 1992 survey (14 stations) was to measure the effects TBT had on dogwhelks, to establish the geographic spread of it around England and Wales and to assess the need for a wider study. Due to the high level of TBT exposure around these coastlines, the network of sites increased from 14 sites in 1992 to 102 in 2007 (Fig. 1, Table 1). These sites include areas close to ports, e.g. Dover, Southampton Water and Milford Haven; areas close to shipping lanes (traffic separation zones), e.g. Dover Straits, Isle of Wight and Holyhead; and areas away from the influence of shipping, including “background reference” sites.

### Sample collection

At each sampling site, up to 100 dogwhelks were collected from the foreshore between spring low water and mid tide levels. The location of the site was recorded using national grid references in the early survey years and in latitude and longitude using the global positioning system (GPS) in more recent years. Date and time of collection were also recorded for ICES reporting purposes.

From the 100 animals collected, 50 specimens (young adults, with toothed shells) were taken back to the laboratory for analysis; animals with worn shells were excluded from the sample as they could be older than 4 years. If no individuals were found within the first 25 min of sampling, the station was abandoned. Each sample was stored in a sealed net bag in a cool box with sea water-wetted seaweed (taken from the sampling site) until the sample was transferred to aquarium facilities in the laboratory. On transfer to these facilities (cool [4–15 °C], aerated and filtered sea water), the animals were stored for as short a period of time as possible before analysis. In general, this was less than 36 h and always less than 7 days.

### Sample processing and quality assurance

Of the 50 individuals brought back from the shore, 40 were chosen at random and analysed. If an individual was parasitised or dead, it was excluded from the analysis and a fresh dogwhelk was added. Prior to the analysis, the length of each dogwhelk was measured to the nearest 0.01 mm with Vernier callipers and then cracked open with a vice for further analysis, which included sex determination according to Fioroni et al. (1991) and Gibbs et al. (1987), penis length measurements in males and females (if present) and the associated imposex stage in females. To identify the stages of imposex, the methods described by Gibbs et al. (1987) were used.

Quality assurance was implemented over the years by ensuring that the analysts have undergone in-house training and participated in ring tests with other organisations, such as Marine Scotland. The analysts also participated in the Quality Assurance of Information for Marine Environmental Monitoring in Europe laboratory proficiency rounds for imposex in marine snails since 2003. The highest possible score of “satisfactory” was achieved every time an analyst participated. The last round for imposex in marine snails took place in 2012. The same analyst of the 2010 and 2014 surveys participated in the 2012 round.

### Calculation of imposex parameter

After all the individuals were measured, the Vas Deferens Sequence Index (VDSI) was calculated as the mean of the Vas Deferens Sequence stages (VDS) observed in one sample to assess the Oslo and Paris Convention (OSPAR) class for each site (Table 2, OSPAR 2004).

**Table 2:** Interpretations of the assessment classes, referring to *Nucella lapillus* used in the Oslo and Paris Convention (OSPAR) monitoring guideline (OSPAR 2004)

OSPAR ass. class	*Nucella lapillus* VDSI	Effects and impacts
A	VDSI = <0.3	The level of imposex in the more sensitive gastropod species is close to 0 (0–∼30 % of females have imposex) indicating exposure to TBT concentrations close to 0, which is the objective in the OSPAR hazardous substances strategy.
B	VDSI = 0.3– <2.0	The level of imposex in the more sensitive gastropod species (∼30–∼100 % of the females have imposex) indicates exposure to TBT concentrations below the Ecotoxicological Assessment Criteria (EAC) derived by OSPAR for TBT, e.g. adverse effects in the more sensitive taxa of the ecosystem caused by long-term exposure to TBT are predicted to be unlikely to occur.
C	VDSI = 2.0– <4.0	The level of imposex in the more sensitive gastropod species indicates exposure to TBT concentrations higher than the EAC derived for TBT, e.g. there is a risk of adverse effects, such as reduced growth and recruitment, in the more sensitive taxa of the ecosystem caused by long-term exposure to TBT.
D	VDSI = 4.0–5.0	The reproductive capacity in the populations of the more sensitive gastropod species, such as *Nucella lapillus*, is affected as a result of the presence of sterile females, but some reproductively capable females remain, e.g. there is evidence of adverse effects, which can be directly associated with the exposure of TBT.
E	VDSI = >5.0	Populations of the more sensitive gastropod species, such as *Nucella lapillus*, are unable to reproduce. The majority, if not all females within the population, have been sterilised.
F	VDSI = -	The populations of the more sensitive gastropod species, such as *Nucella lapillus*, are absent/expired.

*VDSI* Vas Deferens Sequence Index

### Statistical analysis

The data were analysed to see whether there was evidence of a trend in mean VDS (VDSI) over time. Trends were assessed by station to avoid biases due to not all stations being sampled on every survey occasion. The survey years used were 1997, 2004, 2007, 2010 and 2014. Stations were included if they were sampled on at least two survey years. This meant that 88 stations were included and only 15 omitted. The 1992 survey could not be included in the assessment as the original VDS values for each dogwhelk were not available (similar to the paper published by Oliveira et al. 2009).

A non-parametric Mann-Kendall (MK) statistic was used to investigate trend. For trend at a single station, the Mann-Kendall method is used as follows. For each VDS observation (except for those in the final survey), the Mann-Kendall (MK) statistic (Mann 1945; Kendall 1975) is the sum of the number of observations from later surveys that are greater than it (+1 per observation) and the number of observations that are less than it (−1 per observation). This is repeated for all time points and the cumulative sum is calculated. We denote this as the MK statistic.

To assign a *p* value to the observed MK statistic, a two-sided randomisation test (Manly 2006) was used. Under the null hypothesis of no trend, the VDS observations can be randomly permuted (keeping the survey year vector fixed). Then, the MK statistic can be calculated as above. This procedure was carried out 999 times in total to estimate the null distribution of the MK statistic. The number of the absolute values of these observations that are greater than the absolute value of the observed MK statistic is noted (call this BIGGER). Following Manly, the *p* value is calculated as (BIGGER + 1)/1000.

For the trend analysis, we have assumed that the samples taken at each station were a reasonably representative spatial sample and that the VDS values within a station were independent. Because we cannot be sure whether this assumption holds fully, we also looked at trends between 1997 and 2010 but by analysing the mean VDS values at each station—and hence negating the need for independence between samples within a station. We considered the 56 stations that were sampled in both 1997 and 2010. For each station, we calculated the mean level of VDS. We then compared the 56 VDSI sampled in 1997 with the same 56 VDSI sampled in 2010 using a paired (between stations) non-parametric Wilcoxon test. The same approach was carried out for the 6 stations that were sampled in both 1997 and 2014. While this is a less powerful comparison, it allows us to examine the most recent data.

## Results

**Fig. 2:**
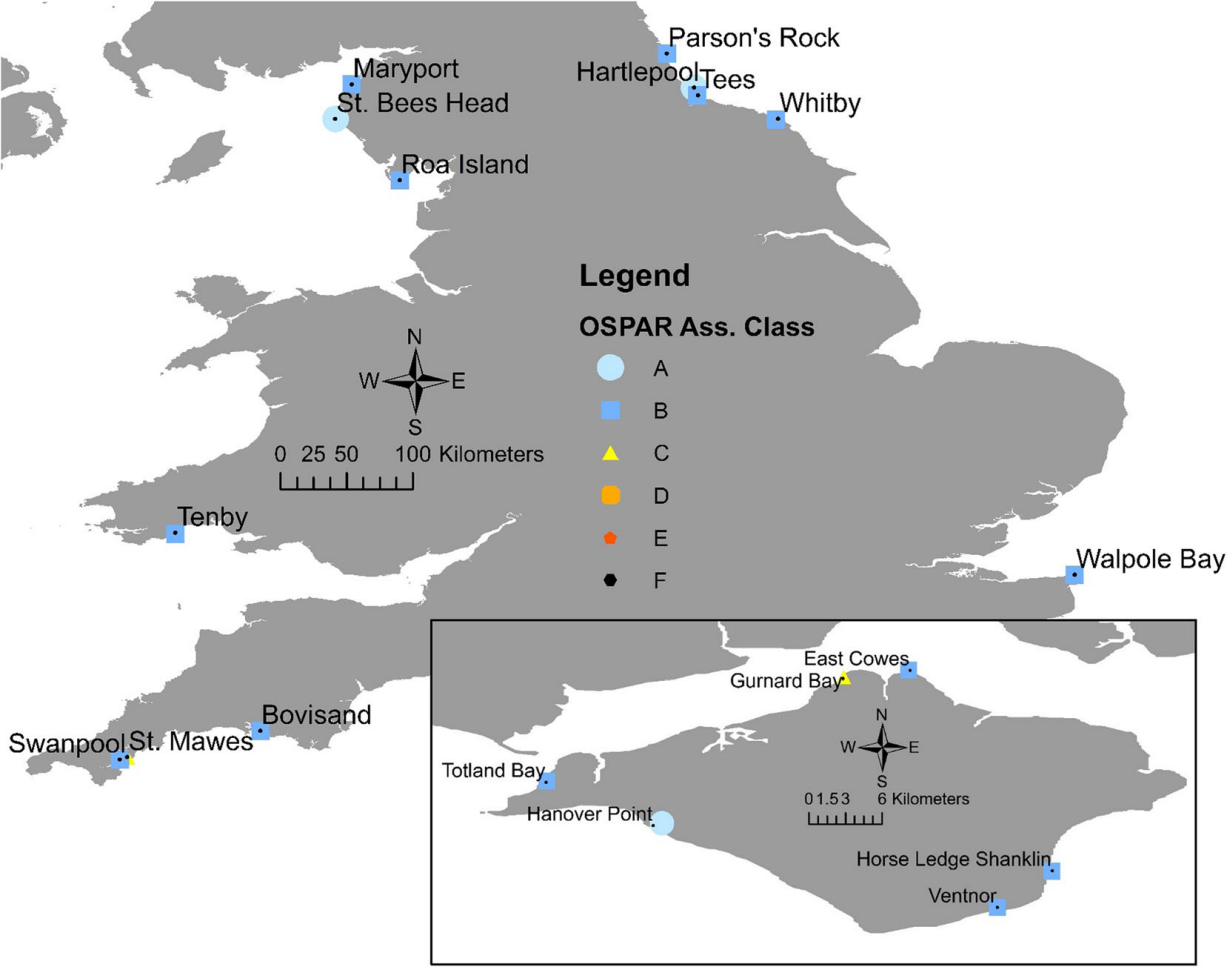
Oslo and Paris Convention classification of dogwhelks from 2014 England and Wales sampling sites; OSPAR classifications go from A (background incidence of imposex) to F (populations of the more sensitive gastropod species are absent/expired)

The results of the 2014 survey show that 2 sites (St. Mawes and Gurnard Bay) still exceed the Ecotoxicological Assessment Criteria (EAC) for TBT exposure with an OSPAR assessment class C, while 3 and 13 sites are at OSPAR assessment class A and B, respectively (Fig. 2 for spatial overview; Table 3 for summary information). Comparing the same 12 sites sampled between 2010 and 2014 shows that three sites (Gurnard Bay, Roa Island and Bovisand) increased in the VDSI (Table S1), with Gurnard Bay and Roa Island also changing to a higher OSPAR assessment class (Table 4). The other nine sites sampled in 2010 and 2014 (Table 1) showed reductions in VDSI with 8 also changing to a lower OSPAR assessment class (Table 4). By comparing the summary results shown in Table 4 of the TBT OSPAR assessment classes, it seemed that there were large decreases in the effects of TBT on *N. lapillus* between 1992 and 2014 in England and Wales.

**Table 3:** Summary results of the 2014 imposex survey separated into strata

Stratum	Site name	Number	VDSI ± St. Dev. 2014	OSPAR ass. class
Anglia	Walpole Bay	18	0.94 ± 1.1	B
Eastern Channel	Hanover Point	22	0.14 ± 0.35	A
Eastern Channel	Horse Ledge Shanklin	25	1.32 ± 1.25	B
Eastern Channel	Ventnor	14	1.07 ± 1.21	B
Eastern Channel	East Cowes^a^	19	0.68 ± 0.82	B
Eastern Channel	Gurnard Bay^a^	14	2.07 ± 1.27	C
Eastern Channel	Totland Bay^a^	28	0.46 ± 0.69	B
Irish Sea	Maryport	20	1.30 ± 1.66	B
Irish Sea	Roa Island^a^	15	0.80 ± 1.01	B
Irish Sea	St. Bees Head^a^	18	0.00 ± 0.00	A
Severn	Tenby	27	1.78 ± 1.55	B
Tyne Tees	Hartlepool	21	0.28 ± 0.56	A
Tyne Tees	Parsons Rock	18	0.72 ± 0.89	B
Tyne Tees	Tees	21	1.38 ± 1.12	B
Tyne Tees	Whitby	22	1.18 ± 1.18	B
Western Channel	St. Mawes	21	2.57 ± 1.36	C
Western Channel	Swanpool	16	1.68 ± 1.45	B
Western Channel	Bovisand^a^	23	0.91 ± 1.00	B

*VDSI* Vas Deferens Sequence Index, *OSPAR ass. class* assessment class as highlighted in Table 2

^a^Six additional sites sampled for the Environment Agency

**Table 4:** *Nucella lapillus* Vas Deferens Sequence Index (VDSI) results since 1992 separated into strata

Stratum	Site name	Latitude	Longitude	Ass. class 1992	Ass. class 1997	Ass. class 2004	Ass. class 2007	Ass. class 2010	Ass. class 2014	MK	*p* value	Trend
Anglia	Dumpton Gap	51.3494	1.442	NA	NA	NA	B	NA	NA	NA	NA	NA
Anglia	Herne Bay	51.3801	1.2	NA	NA	NA	B	NA	NA	NA	NA	NA
Anglia	Palm Bay	51.3934	1.4222	NA	NA	NA	B	A	NA	−59	0.06	↘
Anglia	Walpole Bay	51.3938	1.405	NA	NA	NA	C	NA	B	−290	0.01	↓
Cardigan Bay	Abermawr	51.9713	−5.0831	NA	B	B	B	A	NA	−322	0.03	↓
Cardigan Bay	Aberystwyth	52.4126	−4.0921	NA	B	A	B	B	NA	−225	0.12	↘
Cardigan Bay	Cable Bay	53.2072	−4.5004	NA	B	NA	B	A	NA	−206	0.01	↓
Cardigan Bay	Caernarfon	53.1403	−4.2845	NA	B	NA	NA	NA	NA	NA	NA	NA
Cardigan Bay	Church Bay (Anglesey)	53.3709	−4.5577	NA	C	NA	B	A	NA	−647	0.01	↓
Cardigan Bay	New Quay	52.2175	−4.3602	NA	B	B	B	NA	NA	−204	0.05	↘
Cardigan Bay	Porth Colmon	52.8755	−4.6824	NA	B	A	A	B	NA	−214	0.01	↓
Cardigan Bay	Puffin Island	53.3136	−4.0412	NA	C	NA	NA	NA	NA	NA	NA	NA
Cardigan Bay	Sandy Beach	53.3338	−4.5764	NA	C	C	B	A	NA	−488	0.01	↓
Cardigan Bay	Sarn Bach	52.8116	−4.4987	NA	B	NA	B	NA	NA	21	0.65	↗
Cardigan Bay	South Stack	53.2999	−4.6793	NA	C	NA	B	B	NA	−404	0.01	↓
Cardigan Bay	Trearddur	53.2072	−4.5004	NA	C	NA	B	A	NA	−390	0.01	↓
Cardigan Bay	Trefor	52.9992	−4.4206	NA	B	NA	B	A	NA	−708	0.01	↓
Eastern Channel	Bembridge	50.6889	−1.0713	NA	NA	NA	C	B	NA	−114	0.1	↘
Eastern Channel	Bill of Portland	50.5125	−2.4577	D	F	NA	B	B	NA	−17	0.46	↘
Eastern Channel	Brixham	50.4004	−3.5052	D	D	C	C	B	NA	−1581	0.01	↓
Eastern Channel	East Cowes	50.7663	−1.276	NA	NA	NA	B	NA	B^a^	−7	0.96	↘
Eastern Channel	Eastbourne	50.7583	0.2832	D	D	C	B	B	NA	−1853	0.01	↓
Eastern Channel	Folkestone	51.0849	1.202	D	D	C	C	NA	NA	−284	0.1	↘
Eastern Channel	Gurnard Bay	50.7602	−1.3254	NA	NA	NA	B	B	C^a^	165	0.01	↑
Eastern Channel	Hanover Point	50.6519	−1.4652	NA	NA	NA	C	NA	A	−300	0.01	↓
Eastern Channel	Hayling Island	50.787	−0.9376	C	NA	NA	NA	NA	NA	NA	NA	NA
Eastern Channel	Horse Ledge Shanklin	50.6186	−1.1714	NA	NA	NA	C	C	B	−744	0.01	↓
Eastern Channel	Selsey Bill	50.7297	−0.7761	D	C	NA	B	B	NA	−539	0.01	↓
Eastern Channel	St. Catherine’s Point	50.5758	−1.3	NA	NA	NA	C	B	NA	−60	0.41	↘
Eastern Channel	St. Margaret’s Bay	51.1523	1.3892	NA	NA	NA	C	B	NA	−371	0.01	↓
Eastern Channel	Totland Bay	50.6839	−1.544	NA	NA	NA	B	NA	B^a^	−278	0.01	↓
Eastern Channel	Ventnor	50.5919	−1.2118	NA	NA	NA	C	NA	B	−202	0.01	↓
Eastern Channel	West Bay	50.7113	−2.7714	C	C	B	NA	B	NA	−1155	0.01	↓
Humber/Wash	Sewerby	54.0995	−0.1525	C	C	C	B	NA	NA	−708	0.01	↓
Irish Sea	Cemaes Bay	53.4153	−4.4525	NA	B	B	B	B	NA	−708	0.01	↓
Irish Sea	Cemlyn Bay	53.4136	−4.5145	NA	B	NA	NA	NA	NA	NA	NA	NA
Irish Sea	Great Ormes Head	53.3252	−3.8604	NA	C	NA	C	A	NA	−178	0.02	↓
Irish Sea	Llanbadrig	53.441	−4.45	NA	C	B	NA	A	NA	−562	0.01	↓
Irish Sea	Llanellen	53.4113	−4.2933	NA	B	NA	C	B	NA	−181	0.05	↘
Irish Sea	Maryport	54.7224	−3.5003	NA	C	C	C	NA	B	−447	0.01	↓
Irish Sea	Moelfre	53.353	−4.2346	NA	C	NA	B	A	NA	−309	0.01	↓
Irish Sea	Rhos-on-Sea	53.3085	−3.7367	NA	B	C	C	B	NA	259	0.13	↘
Irish Sea	Roa Island	54.0726	−3.1735	NA	C	B	A	A	B^a^	−490	0.01	↓
Irish Sea	St. Bees Head	54.4898	−3.6122	NA	B	B	A	B	A^a^	−1184	0.01	↓
Irish Sea	Whitehaven	54.5591	−3.5913	NA	B	NA	A	A	NA	−602	0.01	↓
Severn	Angle Bay	51.688	−5.0754	NA	D	NA	C	B	NA	−928	0.01	↓
Severn	Barry Island	51.3858	−3.2783	NA	NA	NA	A	A	NA	−54	0.24	↘
Severn	Combe Martin	51.2091	−4.0414	NA	C	B	A	A	NA	−890	0.01	↓
Severn	Dale Fort	51.7039	−5.1524	NA	D	C	C	B	NA	−1159	0.01	↓
Severn	Freshwater West	51.6536	−5.0611	NA	D	C	C	B	NA	−1092	0.01	↓
Severn	Hartland Quay	50.996	−4.5336	NA	C	B	A	A	NA	−1559	0.01	↓
Severn	Holywell Bay	50.3906	−5.1477	NA	B	NA	NA	NA	NA	NA	NA	NA
Severn	Lavernock Point	51.4054	−3.1669	NA	D	C	B	A	NA	−1582	0.01	↓
Severn	Limpert Bay	51.3834	−3.4144	NA	NA	NA	A	A	NA	−44	0.14	↘
Severn	Manorbier	51.643	−4.8063	NA	C	C	A	A	NA	−1458	0.01	↓
Severn	Marloes Sands	51.7226	−5.2167	NA	C	C	A	A	NA	−1200	0.01	↓
Severn	Martin’s Haven	51.7365	−5.2445	NA	C	B	B	A	NA	−213	0.24	↘
Severn	Monk Haven	51.7097	−4.1454	NA	D	C	C	B	NA	−953	0.01	↓
Severn	Penarth	51.3938	−3.1605	NA	NA	NA	B	NA	NA	NA	NA	NA
Severn	Pennar Jetty	51.6888	−4.9777	NA	C	NA	NA	NA	NA	NA	NA	NA
Severn	Pennar Point	51.6919	−4.9662	NA	C	NA	C	A	NA	−488	0.01	↓
Severn	Perranporth	50.348	−5.1582	NA	B	NA	B	A	NA	−226	0.01	↓
Severn	Polzeath	50.5743	−4.9216	NA	C	C	B	A	NA	−577	0.01	↓
Severn	Porlock Weir	51.2192	−3.6235	NA	C	C	B	B	NA	−1004	0.01	↓
Severn	Port Eynon	51.5408	−4.2063	NA	C	B	B	A	NA	−1457	0.01	↓
Severn	Porth Beach	50.426	−5.0628	NA	C	C	C	B	NA	−813	0.01	↓
Severn	Porthcorthan	50.5098	−5.0303	NA	B	B	A	B	NA	−444	0.01	↓
Severn	Porthtowan	50.2871	−5.2454	NA	B	A	A	A	NA	−363	0.01	↓
Severn	Portquin	50.5897	−4.8694	NA	C	NA	A	A	NA	−531	0.01	↓
Severn	Renny’s Slip	51.7324	−5.2447	NA	C	C	NA	A	NA	−250	0.02	↓
Severn	Sennen Cove	50.0785	−5.7034	NA	C	C	B	B	NA	−1411	0.01	↓
Severn	Stackpole Quay	51.6241	−4.8999	NA	C	C	B	B	NA	−1080	0.01	↓
Severn	Tenby	51.6792	−4.6989	NA	C	C	B	C	B	−312	0.08	↘
Severn	Treknow	50.6452	−4.7624	NA	C	NA	A	A	NA	−425	0.01	↓
Severn	Trenance	50.4656	−5.0344	NA	B	NA	NA	NA	NA	NA	NA	NA
Severn	Trevone	50.5456	−4.981	NA	C	NA	NA	NA	NA	NA	NA	NA
Severn	Watwick Bay	51.6921	−5.1601	NA	D	C	C	B	NA	−1294	0.01	↓
Severn	West Angle Bay	51.6859	−5.1102	NA	D	C	C	B	NA	−1186	0.01	↓
Severn	West Pentire	50.4056	−5.131	NA	C	NA	NA	NA	NA	NA	NA	NA
Severn	Westdale Bay	51.7073	−5.1881	NA	C	C	B	A	NA	−1623	0.01	↓
Severn	Whitesands Bay	51.898	−5.2986	NA	B	C	B	NA	NA	−280	0.07	↘
Tyne Tees	Amble	55.3377	−1.5714	NA	NA	C	NA	A	NA	−128	0.01	↓
Tyne Tees	Blyth	55.1306	−1.5001	NA	NA	C	B	B	NA	−576	0.01	↓
Tyne Tees	Blyth Ferry	55.1242	−1.4892	C	C	NA	NA	NA	NA	NA	NA	NA
Tyne Tees	Boulmer	55.4241	−1.5774	C	C	B	B	A	NA	−1747	0.01	↓
Tyne Tees	Hartlepool	54.7	−1.1794	NA	NA	C	B	C	A	−901	0.01	↓
Tyne Tees	Parson’s Rock	54.9316	−1.3659	NA	NA	NA	C	D	B	−336	0.01	↓
Tyne Tees	Robin Hood’s Bay	54.4232	−0.5277	NA	NA	NA	B	B	NA	96	0.17	↘
Tyne Tees	Saltburn	54.5869	−0.951	NA	NA	NA	B	B	NA	6	0.94	↗
Tyne Tees	Scarborough	54.2881	−0.3957	NA	NA	NA	C	B	NA	−190	0.01	↓
Tyne Tees	St. Mary’s Lighthouse	55.0717	−1.4525	NA	NA	C	NA	B	NA	−436	0.01	↓
Tyne Tees	Tees	54.648	−1.1534	NA	NA	NA	C	C	B	−465	0.01	↓
Tyne Tees	Tynemouth (Outside)	55.019	−1.4176	NA	NA	NA	C	B	NA	−67	0.13	↘
Tyne Tees	Whitby	54.4907	−0.6093	D	D	C	C	C	B	−1473	0.01	↓
Tyne Tees	Whitley Bay	55.039	−1.4312	NA	NA	NA	C	B	NA	−78	0.02	↓
Western Channel	Bovisand	50.3349	−4.1208	D	D	C	B	B	B^a^	−2652	0.01	↓
Western Channel	Gwennap Head	50.0367	−5.6716	C	C	C	B	A	NA	−1812	0.01	↓
Western Channel	St. Agnes	50.3222	−5.2043	NA	B	NA	NA	NA	NA	NA	NA	NA
Western Channel	St. Mawes	50.1554	−5.0251	NA	NA	NA	C	C	C	−120	0.32	↘
Western Channel	Start Point	50.2197	−3.6501	D	C	C	B	A	NA	−1866	0.01	↓
Western Channel	Swanpool	50.1396	−5.0763	NA	NA	NA	C	C	B	−248	0.07	↘
Western Channel	Towan Head	50.1563	−4.984	NA	C	NA	C	B	NA	−101	0.3	↘

A trend indication of the VDSI shows if a site either changed statistically significantly (↓↑) or not significantly (↘↗) within surveys between 1997 and 2014

*NA* no data available at this site or was not sampled or no adult females were found, − no *N. lapillus* found due to extinction, *Ass. class* assessment class as highlighted in Table 2, *MK* Mann-Kendall

^a^Six additional sites sampled for the Environment Agency

While recognising that comparisons between years are not strictly appropriate because the same stations are not always sampled in the same year, it is interesting to see how the percentages in the various OSPAR assessment classes changed over years. Not one station out of 14 of the 1992 England and Wales survey was at background imposex level, class A on the OSPAR assessment scale, whereas 100 % of stations were at classes C or D. In contrast, in 2010, 43 % of the 77 sites were class A, followed by 47, 9 and 1 % for B, C and D, respectively. In the intervening surveys, 1997, 2004 and 2007, there was a gradual improvement in the level of imposex in each class recorded as shown by the changes in the proportion of imposex within each class (Table 5). The summary statistics of the imposex development in England and Wales between 1992 and 2014 indicate an increase in the number of sites at OSPAR assessment classes A and B (from 0 % for A and B in 1992 to 17 and 72 % in 2014, respectively; Table 5).

**Table 5:** Percentage of sites in each OSPAR assessment class for imposex for each reporting year between 1992 and 2014 for England and Wales

		% of sites in each OSPAR ass. class					
Sampling year	Sampling sites	F	E	D	C	B	A
1992	14	0	0	57	43	0	0
1997	72	1	0	17	53	29	0
2004	48	0	0	0	67	27	6
2007	86	0	0	0	35	49	16
2010	77	0	0	1	9	47	43
2014	18	0	0	0	11	72	17

*OSPAR ass. class* Oslo and Paris Convention assessment class as highlighted in Table 2

A VDSI trend assessment between the 1997 and 2014 surveys was carried out with the non-parametric Mann-Kendall statistic. The results highlighted in Table 4 show that 65 sites out of 88 reduced statistically significantly in VDSI. The Gurnard Bay site showed a significant increase in VDSI. Twenty sites showed not significant reductions and two not significant increases in VDSI (Table 4). A direct comparison of the same 56 sites sampled in the 1997 and 2010 surveys showed that the VDSI reduced from 2.89 and 0.42, respectively—this difference was strongly statistically significant (*p* > 0.001). Another comparison of the same 6 sites sampled in 1997 and 2014 showed a statistically significant VDSI reduction from 3.14 in 1997 to 1 in 2014 (*p* = 0.03). The summary information of the observed VDSI can be found in Table S1 in the supplementary section.

## Discussion

Over the past two decades of regular surveys, there has been a consistent decrease in imposex levels corresponding to the implementation of different legislation controlling the use of TBT (Birchenough et al. 2002; Bryan et al. 1993). The results described in the 2014 survey show a continued decline in the incidence of imposex since the 1997 survey when looking at the same 6 stations.

The decline has been most marked in 2007 and 2010, probably as a result of the phasing out and complete supposed removal of TBT antifouling paints from boats on 17 September 2008. These results are similar to a study for sites around Portugal by Galante-Oliveira et al. (2011). A general decrease was observed in Portugal: whereas 56 % of dogwhelks had a VDSI value of 2 or higher in 2006, in 2008, only 17 % showed a similar level of effect. This correlates well with our study, where 35 % of dogwhelks had VDSI values greater than 2 in 2007 and only 10 % in 2010. The OSPAR monitoring community also observed a downward trend in VDSI. Over 80 % of sites showing a downward trend and 16 % showed a non-improvement, while 4 % show a stable background concentration level (OSPAR 2014).

Currently, the only source of TBT in the marine environment is from historical/residual contamination in sediments in estuaries and harbours (Sousa et al. 2009) and arising from dredged material disposal, but this activity is tightly controlled and is unlikely to be a significant source of contamination in the future as disposal sites are chosen to be dispersive and insuring that sediments will not stay on one site within England and Wales. Nevertheless, two sites (St. Mawes and Gurnard Bay) measured OSPAR assessment class C in 2014, failing the EAC that was derived for TBT, meaning that there is a risk of adverse effects, such as reduced growth and recruitment (OSPAR 2004). Langston et al. (2015) observed a VDSI of 2.76 in St. Mawes in 2012 which is between the 2010 and 2014 survey result of these surveys (3.73 and 2.57, respectively). Currently, it is difficult to understand why there has not been a significant decrease at these sites. Local illegal use of TBT antifouling paint cannot be ruled out, but it is more likely that historic pollution in sediments may act as a source of TBT for a longer time period in certain areas (OSPAR 2013b). Gibbs (2009) observed little improvement in the reproductive capacity of the population at a shipyard near Falmouth. The TBT pollution in his study area was probably a result of discharges from the shipyards or release or re-suspension from sediments. This could be another explanation for the continuing TBT impacts. His results correlate with the constantly high levels at St. Mawes station Falmouth in our study. As imposex is irreversible in dogwhelks (Bryan et al. 1986) and since they live for up to 7 years, it will take time for the most affected animals (class C and above) to disappear from the population. Nevertheless, clear evidence that dogwhelk populations are recovering at all sites is shown by the presence of juveniles and eggs recorded at each site. Eggs were present at many of the sites, but juveniles were observed at all 77 sites in the 2010 survey and all 18 sites in the 2014 survey. Because dogwhelks do not have a pelagic dispersal phase in their life cycle, it indicates that all populations are actively and successfully reproducing. In 1997, *N. lapillus* was deemed extinct from the Bill of Portland, but due to the ability to re-colonise areas where extinction has occurred (Huet et al. 2004), these organisms were observed again from 2007 onwards.

## Conclusions

Imposex in dogwhelks was attributed to TBT-based antifouling paints used on yachts and ships in the UK in the 1980s (Gibbs and Bryan 1986). Since then, the UK Government has actively pursued legislation to control the use of TBT in the marine environment and supported UK-wide marine monitoring surveys of imposex. As a result, the evidence shown here clearly demonstrates the efficacy of the policy in improving dogwhelk health and in many England and Wales foreshores. The data set shows a good example of science and policy working hand-in-hand for environmental benefit. The continued measurement of imposex in marine molluscs offers the most clear-cut tool for monitoring of a contaminant-specific pollution effect under the Marine Strategy Framework Directive (OSPAR 2014).

## Electronic supplementary material

ESM 1 (DOCX 35 kb)
