# Supplementary material for: Imposex in the dogwhelk (Nucella lapillus): 22-year monitoring around England and Wales
Source: Environ Monit Assess. 2015 Nov 10;187:736. doi: 10.1007/s10661-015-4961-0 (PMC4641150; doi:10.1007/s10661-015-4961-0)
Supplement: Supplementary file 1 — (DOCX 35 kb) [file 10661_2015_4961_MOESM1_ESM.docx]

Table S1. Summary overview of the observed dogwhelk *Nucella lapillus’* Vas Deferens Sequence Index (VDSI) and the associated Standard Deviation (St. Dev.) at each station. N represents the number of females sampled at each station. The 1992 survey does not have any additional information as the individual Vas Deferens Sequence stages (VDS) data was not available. - the dogwhelk was extinct from the sampling site.

| Stratum | Site Name | VDSI 1992 | VDSI 1997 | St. Dev. 1997 | N 1997 | VDSI 2004 | St. Dev. 2004 | N 2004 | VDSI 2007 | St. Dev. 2007 | N 2007 | VDSI 2010 | St. Dev. 2010 | N 2010 | VDSI 2014 | St.Dev. 2014 | N 2014 |
| --- | --- | --- | --- | --- | --- | --- | --- | --- | --- | --- | --- | --- | --- | --- | --- | --- | --- |
| Anglia | Dumpton Gap | NA | NA |  |  | NA |  |  | 1.21 | 1.85 | 14 | NA |  |  | NA |  |  |
| Anglia | Herne Bay | NA | NA |  |  | NA |  |  | 1.63 | 2.00 | 8 | NA |  |  | NA |  |  |
| Anglia | Palm Bay | NA | NA |  |  | NA |  |  | 1.72 | 1.64 | 18 | 0.29 | 0.49 | 7 | NA |  |  |
| Anglia | Walpole Bay | NA | NA |  |  | NA |  |  | 3.25 | 1.02 | 20 | NA |  |  | 0.94 | 1.11 | 18 |
| Cardigan Bay | Abermawr | NA | 1.25 | 1.00 | 16 | 1.95 | 1.19 | 20 | 1.50 | 1.45 | 12 | 0.00 | 0.00 | 11 | NA |  |  |
| Cardigan Bay | Aberystwyth | NA | 0.80 | 0.86 | 15 | 0.20 | 0.52 | 20 | 0.53 | 1.07 | 17 | 0.31 | 0.70 | 16 | NA |  |  |
| Cardigan Bay | Cable Bay | NA | 1.47 | 1.18 | 17 | NA |  |  | 0.81 | 1.17 | 16 | 0.14 | 0.38 | 7 | NA |  |  |
| Cardigan Bay | Caernarfon | NA | 0.50 | 1.00 | 4 | NA |  |  | NA |  |  | NA |  |  | NA |  |  |
| Cardigan Bay | Church Bay (Anglesey) | NA | 2.44 | 1.04 | 18 | NA |  |  | 1.17 | 1.61 | 24 | 0.06 | 0.24 | 17 | NA |  |  |
| Cardigan Bay | New Quay | NA | 1.82 | 1.30 | 22 | 1.31 | 1.30 | 16 | 1.00 | 1.11 | 14 | NA |  |  | NA |  |  |
| Cardigan Bay | Porth Colmon | NA | 1.21 | 1.05 | 14 | 0.27 | 0.65 | 11 | 0.00 | 0.00 | 11 | 0.40 | 0.70 | 10 | NA |  |  |
| Cardigan Bay | Puffin Island | NA | 2.13 | 0.83 | 15 | NA |  |  | NA |  |  | NA |  |  | NA |  |  |
| Cardigan Bay | Sandy Beach | NA | 2.57 | 0.76 | 14 | 2.10 | 1.07 | 20 | 1.67 | 1.56 | 12 | 0.00 | 0.00 | 9 | NA |  |  |
| Cardigan Bay | Sarn Bach | NA | 1.68 | 0.95 | 19 | NA |  |  | 1.90 | 1.20 | 10 | NA |  |  | NA |  |  |
| Cardigan Bay | South Stack | NA | 2.36 | 1.08 | 25 | NA |  |  | 1.06 | 1.26 | 18 | 0.71 | 0.76 | 7 | NA |  |  |
| Cardigan Bay | Trearddur | NA | 2.27 | 0.70 | 15 | NA |  |  | 0.74 | 1.10 | 19 | 0.20 | 0.63 | 10 | NA |  |  |
| Cardigan Bay | Trefor | NA | 1.95 | 1.22 | 19 | NA |  |  | 0.75 | 1.36 | 24 | 0.00 | 0.00 | 19 | NA |  |  |
| Eastern Channel | Bembridge | NA | NA |  |  | NA |  |  | 2.56 | 1.10 | 18 | 1.68 | 1.53 | 19 | NA |  |  |
| Eastern Channel | Bill of Portland | 4.00 | - |  | 0 | NA |  |  | 0.41 | 0.51 | 17 | 0.67 | 1.15 | 3 | NA |  |  |
| Eastern Channel | Brixham | 4.33 | 4.00 | 0.65 | 20 | 3.65 | 0.49 | 20 | 3.11 | 1.02 | 18 | 1.38 | 1.13 | 24 | NA |  |  |
| Eastern Channel | East Cowes | NA | NA |  |  | NA |  |  | 1.19 | 1.72 | 16 | NA |  |  | 0.68 | 0.82 | 19 |
| Eastern Channel | Eastbourne | 4.10 | 4.08 | 0.64 | 25 | 3.76 | 0.54 | 21 | 0.88 | 1.23 | 24 | 0.36 | 0.50 | 14 | NA |  |  |
| Eastern Channel | Folkestone | 4.36 | 4.03 | 0.68 | 29 | 3.63 | 0.49 | 24 | 3.58 | 1.02 | 19 | NA |  |  | NA |  |  |
| Eastern Channel | Gurnard Bay | NA | NA |  |  | NA |  |  | 1.27 | 1.62 | 15 | 1.58 | 1.62 | 12 | 2.07 | 1.27 | 14 |
| Eastern Channel | Hanover Point | NA | NA |  |  | NA |  |  | 2.10 | 1.71 | 20 | NA |  |  | 0.14 | 0.35 | 22 |
| Eastern Channel | Hayling Island | 2.09 | NA |  |  | NA |  |  | NA |  |  | NA |  |  | NA |  |  |
| Eastern Channel | Horse Ledge Shanklin | NA | NA |  |  | NA |  |  | 3.35 | 0.83 | 23 | 2.36 | 1.34 | 14 | 1.32 | 1.25 | 25 |
| Eastern Channel | Selsey Bill | 4.14 | 3.90 | 0.65 | 31 | NA |  |  | 0.64 | 0.92 | 11 | 0.86 | 1.21 | 7 | NA |  |  |
| Eastern Channel | St. Catherine's Point | NA | NA |  |  | NA |  |  | 2.00 | 1.52 | 21 | 1.68 | 1.25 | 22 | NA |  |  |
| Eastern Channel | St. Margarets Bay | NA | NA |  |  | NA |  |  | 2.62 | 1.58 | 26 | 0.78 | 0.90 | 23 | NA |  |  |
| Eastern Channel | Totland Bay | NA | NA |  |  | NA |  |  | 1.76 | 1.55 | 21 | NA |  |  | 0.46 | 0.69 | 28 |
| Eastern Channel | Ventnor | NA | NA |  |  | NA |  |  | 3.39 | 1.04 | 18 | NA |  |  | 1.07 | 1.21 | 14 |
| Eastern Channel | West Bay | 3.97 | 3.00 | 0.71 | 29 | 1.05 | 1.05 | 20 | NA |  |  | 0.83 | 0.89 | 23 | NA |  |  |
| Humber/Wash | Sewerby | 3.88 | 3.86 | 0.71 | 22 | 2.30 | 0.47 | 20 | 1.00 | 1.33 | 10 | NA |  |  | NA |  |  |
| Irish Sea | Cemaes Bay | NA | 1.63 | 1.01 | 19 | 1.15 | 1.31 | 20 | 0.62 | 0.74 | 21 | 0.38 | 0.96 | 13 | NA |  |  |
| Irish Sea | Cemlyn Bay | NA | 1.67 | 0.91 | 18 | NA |  |  | NA |  |  | NA |  |  | NA |  |  |
| Irish Sea | Great Ormes Head | NA | 2.83 | 0.82 | 24 | NA |  |  | 2.06 | 1.39 | 18 | 0.00 | * | 1 | NA |  |  |
| Irish Sea | Llanbadrig | NA | 3.35 | 0.83 | 23 | 1.05 | 1.10 | 20 | NA |  |  | 0.20 | 0.45 | 5 | NA |  |  |
| Irish Sea | Llanelllen | NA | 1.87 | 1.10 | 23 | NA |  |  | 2.27 | 1.56 | 11 | 0.50 | 1.17 | 12 | NA |  |  |
| Irish Sea | Maryport | NA | 3.90 | 0.32 | 10 | 2.36 | 1.34 | 14 | 2.29 | 1.80 | 7 | NA |  |  | 1.30 | 1.66 | 20 |
| Irish Sea | Moelfre | NA | 2.47 | 1.22 | 19 | NA |  |  | 0.38 | 0.72 | 16 | 0.00 | 0.00 | 3 | NA |  |  |
| Irish Sea | Rhos-an-Sea | NA | 1.77 | 0.91 | 26 | 2.55 | 1.23 | 20 | 2.44 | 1.15 | 18 | 0.67 | 0.58 | 3 | NA |  |  |
| Irish Sea | Roa Island | NA | 3.00 | 0.77 | 11 | 1.79 | 1.48 | 14 | 0.06 | 0.25 | 16 | 0.00 | * | 1 | 0.80 | 1.01 | 15 |
| Irish Sea | St. Bees Head | NA | 1.50 | 1.02 | 14 | 1.43 | 1.16 | 21 | 0.21 | 0.54 | 19 | 0.38 | 0.52 | 8 | 0.00 | 0.00 | 18 |
| Irish Sea | Whitehaven | NA | 1.96 | 0.56 | 23 | NA |  |  | 0.19 | 0.40 | 21 | 0.00 | 0.00 | 5 | NA |  |  |
| Severn | Angle Bay | NA | 4.33 | 0.64 | 24 | NA |  |  | 3.73 | 0.94 | 22 | 0.72 | 1.13 | 18 | NA |  |  |
| Severn | Barry Island | NA | NA |  |  | NA |  |  | 0.24 | 0.89 | 21 | 0.00 | 0.00 | 27 | NA |  |  |
| Severn | Coombe Martin | NA | 2.36 | 1.25 | 25 | 1.14 | 1.36 | 22 | 0.00 | 0.00 | 9 | 0.00 | 0.00 | 10 | NA |  |  |
| Severn | Dale Fort | NA | 4.75 | 0.75 | 12 | 3.57 | 0.60 | 21 | 2.63 | 1.46 | 19 | 0.73 | 1.28 | 15 | NA |  |  |
| Severn | Freshwater West | NA | 4.29 | 0.46 | 21 | 3.68 | 0.48 | 19 | 2.61 | 1.58 | 18 | 1.33 | 1.37 | 12 | NA |  |  |
| Severn | Hartland Quay | NA | 2.26 | 1.14 | 23 | 0.79 | 1.06 | 24 | 0.24 | 0.97 | 17 | 0.04 | 0.20 | 25 | NA |  |  |
| Severn | Holywell Bay | NA | 1.88 | 1.26 | 16 | NA |  |  | NA |  |  | NA |  |  | NA |  |  |
| Severn | Lavernock Point | NA | 4.00 | 0.00 | 16 | 2.55 | 0.83 | 20 | 0.32 | 0.78 | 22 | 0.00 | 0.00 | 14 | NA |  |  |
| Severn | Limpert Bay | NA | NA |  |  | NA |  |  | 0.17 | 0.51 | 18 | 0.00 | 0.00 | 22 | NA |  |  |
| Severn | Manorbier | NA | 2.81 | 0.81 | 21 | 2.20 | 1.01 | 20 | 0.00 | 0.00 | 20 | 0.00 | 0.00 | 13 | NA |  |  |
| Severn | Marloes Sands | NA | 2.26 | 0.87 | 19 | 2.35 | 1.53 | 23 | 0.18 | 0.66 | 22 | 0.25 | 0.45 | 16 | NA |  |  |
| Severn | Martin's Haven | NA | 2.00 | 1.20 | 19 | 1.00 | 1.03 | 20 | 1.69 | 1.56 | 29 | 0.00 | 0.00 | 2 | NA |  |  |
| Severn | Monk Haven | NA | 4.29 | 0.59 | 17 | 3.00 | 1.26 | 20 | 2.33 | 1.62 | 21 | 0.80 | 1.48 | 10 | NA |  |  |
| Severn | Penarth | NA | NA |  |  | NA |  |  | 0.67 | 0.91 | 21 | NA |  |  | NA |  |  |
| Severn | Pennar Jetty | NA | 3.05 | 1.09 | 22 | NA |  |  | NA |  |  | NA |  |  | NA |  |  |
| Severn | Pennar Point | NA | 3.88 | 0.82 | 26 | NA |  |  | 3.54 | 0.88 | 13 | 0.18 | 0.60 | 11 | NA |  |  |
| Severn | Perranporth | NA | 1.42 | 0.96 | 19 | NA |  |  | 0.71 | 1.14 | 14 | 0.00 | 0.00 | 6 | NA |  |  |
| Severn | Polzeath | NA | 3.50 | 1.00 | 20 | 2.95 | 1.32 | 21 | 0.92 | 1.50 | 13 | 0.00 | 0.00 | 2 | NA |  |  |
| Severn | Porlock Weir | NA | 2.56 | 0.71 | 25 | 2.00 | 1.51 | 22 | 1.10 | 1.60 | 10 | 0.39 | 0.70 | 18 | NA |  |  |
| Severn | Port Eynon | NA | 2.80 | 1.01 | 20 | 1.55 | 0.94 | 20 | 0.68 | 0.99 | 22 | 0.00 | 0.00 | 15 | NA |  |  |
| Severn | Porth Beach | NA | 3.80 | 0.56 | 15 | 3.63 | 0.88 | 27 | 3.24 | 0.95 | 29 | 0.86 | 1.21 | 7 | NA |  |  |
| Severn | Porthcorthan | NA | 1.92 | 1.26 | 13 | 0.33 | 0.86 | 21 | 0.16 | 0.37 | 19 | 0.36 | 0.50 | 11 | NA |  |  |
| Severn | Porthtowan | NA | 1.20 | 1.32 | 15 | 0.15 | 0.49 | 20 | 0.00 | 0.00 | 10 | 0.00 | 0.00 | 9 | NA |  |  |
| Severn | Portquin | NA | 2.19 | 0.75 | 16 | NA |  |  | 0.13 | 0.50 | 16 | 0.00 | 0.00 | 17 | NA |  |  |
| Severn | Renny's Slip | NA | 2.86 | 1.17 | 22 | 2.00 | 1.52 | 20 | NA |  |  | 0.00 | 0.00 | 3 | NA |  |  |
| Severn | Sennen Cove | NA | 2.92 | 1.09 | 26 | 2.35 | 0.93 | 20 | 0.50 | 1.15 | 18 | 0.86 | 1.06 | 21 | NA |  |  |
| Severn | Stackpole Quay | NA | 2.64 | 0.90 | 22 | 2.00 | 1.30 | 20 | 1.37 | 1.46 | 19 | 0.38 | 0.81 | 16 | NA |  |  |
| Severn | Tenby | NA | 2.25 | 0.75 | 12 | 3.24 | 0.83 | 17 | 0.56 | 0.88 | 9 | 2.00 | * | 1 | 1.78 | 1.55 | 27 |
| Severn | Trenance | NA | 0.88 | 0.93 | 17 | NA |  |  | NA |  |  | NA |  |  | NA |  |  |
| Severn | Trevone | NA | 2.83 | 1.20 | 24 | NA |  |  | NA |  |  | NA |  |  | NA |  |  |
| Severn | Treknow | NA | 2.12 | 1.27 | 25 | NA |  |  | 0.00 | 0.00 | 11 | 0.00 | 0.00 | 6 | NA |  |  |
| Severn | Watwick Bay | NA | 4.31 | 0.55 | 26 | 3.15 | 0.88 | 20 | 2.93 | 1.22 | 15 | 1.08 | 1.32 | 13 | NA |  |  |
| Severn | West Angle Bay | NA | 4.33 | 0.49 | 15 | 3.90 | 0.31 | 20 | 2.65 | 1.31 | 20 | 0.58 | 1.00 | 12 | NA |  |  |
| Severn | West Pentire | NA | 2.42 | 1.12 | 19 | NA |  |  | NA |  |  | NA |  |  | NA |  |  |
| Severn | Westdale Bay | NA | 3.76 | 0.60 | 25 | 3.82 | 0.50 | 22 | 0.74 | 1.37 | 19 | 0.18 | 0.53 | 17 | NA |  |  |
| Severn | Whitesands Bay | NA | 1.94 | 1.03 | 17 | 2.14 | 1.39 | 21 | 1.00 | 1.29 | 19 | NA |  |  | NA |  |  |
| Tyne Tees | Amble | NA | NA |  |  | 2.50 | 1.36 | 20 | NA |  |  | 0.25 | 0.46 | 8 | NA |  |  |
| Tyne Tees | Blyth | NA | NA |  |  | 3.54 | 0.78 | 13 | 0.44 | 0.71 | 25 | 0.37 | 0.60 | 19 | NA |  |  |
| Tyne Tees | Blyth Ferry | 3.96 | 3.86 | 0.56 | 22 | NA |  |  | NA |  |  | NA |  |  | NA |  |  |
| Tyne Tees | Boulmer | 3.97 | 3.52 | 0.64 | 27 | 0.90 | 1.02 | 20 | 0.77 | 1.07 | 22 | 0.29 | 0.47 | 17 | NA |  |  |
| Tyne Tees | Hartlepool | NA | NA |  |  | 3.63 | 1.02 | 16 | 1.56 | 1.72 | 18 | 2.00 | 1.26 | 11 | 0.28 | 0.56 | 21 |
| Tyne Tees | Parson's Rock | NA | NA |  |  | NA |  |  | 3.55 | 1.15 | 20 | 4.00 | * | 1 | 0.72 | 0.89 | 18 |
| Tyne Tees | Robin Hood's Bay | NA | NA |  |  | NA |  |  | 1.26 | 1.63 | 23 | 1.92 | 0.90 | 12 | NA |  |  |
| Tyne Tees | Saltburn | NA | NA |  |  | NA |  |  | 0.85 | 1.35 | 20 | 0.86 | 1.21 | 7 | NA |  |  |
| Tyne Tees | Scarborough | NA | NA |  |  | NA |  |  | 2.95 | 1.50 | 20 | 1.47 | 1.01 | 17 | NA |  |  |
| Tyne Tees | St. Mary's Lighthouse | NA | NA |  |  | 3.20 | 0.77 | 20 | NA |  |  | 0.78 | 0.85 | 23 | NA |  |  |
| Tyne Tees | Tees | NA | NA |  |  | NA |  |  | 2.80 | 1.50 | 25 | 2.46 | 1.33 | 13 | 1.38 | 1.12 | 21 |
| Tyne Tees | Tynemouth (Outside) | NA | NA |  |  | NA |  |  | 2.20 | 1.67 | 20 | 1.27 | 1.49 | 11 | NA |  |  |
| Tyne Tees | Whitby | 4.04 | 4.16 | 0.62 | 25 | 2.71 | 1.73 | 14 | 2.15 | 1.57 | 20 | 2.00 | * | 1 | 1.18 | 1.18 | 22 |
| Tyne Tees | Whitley Bay | NA | NA |  |  | NA |  |  | 3.19 | 1.47 | 16 | 1.67 | 1.22 | 9 | NA |  |  |
| Western Channel | Bovisand | 4.69 | 4.03 | 0.48 | 31 | 2.20 | 1.67 | 20 | 1.26 | 1.57 | 23 | 0.50 | 0.89 | 16 | 0.91 | 1.00 | 23 |
| Western Channel | Gwennap Head | 3.74 | 3.71 | 0.47 | 17 | 2.35 | 0.67 | 20 | 1.00 | 1.21 | 27 | 0.06 | 0.25 | 16 | NA |  |  |
| Western Channel | St. Agnes | NA | 1.71 | 1.00 | 24 | NA |  |  | NA |  |  | NA |  |  | NA |  |  |
| Western Channel | St. Mawes | NA | NA |  |  | NA |  |  | 2.59 | 1.80 | 17 | 3.73 | 0.47 | 11 | 2.57 | 1.36 | 21 |
| Western Channel | Start Point | 4.00 | 3.77 | 0.69 | 22 | 3.08 | 1.10 | 24 | 1.11 | 1.49 | 19 | 0.00 | 0.00 | 19 | NA |  |  |
| Western Channel | Swanpool | NA | NA |  |  | NA |  |  | 2.78 | 1.51 | 23 | 2.14 | 1.56 | 14 | 1.68 | 1.45 | 16 |
| Western Channel | Towan Head | NA | 2.31 | 1.03 | 13 | NA |  |  | 2.45 | 1.43 | 20 | 1.50 | 1.35 | 10 | NA |  |  |
